# Epidemiological Characteristics and Factors Associated with Repeat Sexually Transmitted Infections in Barcelona, Spain Over a Decade

**DOI:** 10.1007/s10508-023-02711-6

**Published:** 2023-10-24

**Authors:** Constanza Jacques-Aviñó, Miguel Alarcón Guitiérrez, María Jesús Barbera, Irene Fuertes, Gemma Martin-Ezquerra, Joaquín Lopez-Contreras, Álvaro Vives, Raquel Rodriguez, Miriam Ros, Cristina Rius, Patricia Garcia de Olalla

**Affiliations:** 1 Servei d’Epidemiologia, Agència de Salut Pública de Barcelona, Barcelona, Spain; 2 Fundació Institut Universitari per a la Recerca a l’Atenció Primària de Salut Jordi Gol i Gurina, Gran Via Corts Catalanes, 587, àtic, 08007 Barcelona, Spain; 3 Universitat Autònoma de Barcelona, Bellaterra, Cerdanyola del Vallès (Barcelona), Bellaterra, Spain; 4 Consorcio de Investigación Biomédica en Red de Epidemiología y Salud Pública, Madrid, Spain; 5 Department of Infectious Diseases, Hospital Universitari Vall d’Hebron, Barcelona, Spain; 6 Dermatology Department, Hospital Clinic, Barcelona, Spain; 7 Dermatology Department, Institut Hospital del Mar d’Investigacions Mèdiques, Hospital del Mar, Barcelona, Spain; 8 Infectious Diseases Unit-Internal Medicine Department, Hospital de la Santa Creu i Sant Pau, Universitat Autonoma de Barcelona, Barcelona, Spain; 9 Fundació Puigvert, Barcelona, Spain; 10 Institut de Recerca de l’Hospital de la Santa Creu i Sant Pau, Barcelona, Spain; 11 Universitat Pompeu Fabra, Barcelona, Spain

**Keywords:** Sexually transmitted infection, Repeated infection, Gonorrhea, Syphilis, Lymphogranuloma venereum

## Abstract

In the last few years, the frequency of sexually transmitted infections (STI) has increased, as has the number of people with multiple infections. The aim of our study was to describe the epidemiological characteristics of persons with repeated bacterial STI and to determine the risk factors for these episodes in persons living in Barcelona during the period 2007–2018. We studied all cases of bacterial STI included in the STI registry of Barcelona. Repeated STI were defined as a diagnosis of gonorrhea, syphilis, or lymphogranuloma venereum (LGV) after a first episode of one of these infections. Analysis was stratified by sex and place of birth. The factors associated with time to reinfection were determined by Kaplan–Meier estimates, while the factors associated with risk of infection were determined by a Cox proportional hazards model. Of 9927 persons with a diagnosis of bacterial STI, 1690 (17.0%) had at least two episodes of STI during the study period. On multivariate analysis, repeat STI were independently associated with male sex assigned at birth (HR: 3.45; 95%CI 2.22–5.36), age less than 34 years (HR: 1.22; 95%CI 1.10–1.35); gay, bisexual, and other men who have sex with men, and transgender o transsexual woman (GBSMS/Trans) (HR: 4.03; 95%CI 3.24–5.03), having gonorrhea as first diagnosis (HR:1.49, 95%CI 1.34–1.66) or LGV (HR:1.75; 95%CI 1.47–2.08) and coinfection with HIV (HR:1.98; 95%CI 1.78–2.21). Sexual health programs should be strengthened to prevent STI and reinfection in key populations.

## Introduction

In the last few years, sexually transmitted infections (STI) have increased both in Spain and elsewhere. In Spain, records show that cases of gonorrhea have increased sixfold (12,359 cases), while cases of syphilis have doubled (5822) in the last 10 years. The Ministry of Health has data for only 4 years but, during this time, cases of lymphogranuloma venereum have increased by 82% (453 infections) (Unidad de vigilancia del VIH y conductas de riesgo, 2017). This upward trend follows a similar pattern in the city of Barcelona, but with higher rates than the Spanish average (Carrere et al., 2021). In Barcelona, this increase has occurred particularly among men who have sex with men (MSM) (Salut & Barcelona, 2016). The increment has been less evident among women, mainly because a high percentage of cases are asymptomatic and because STI screening is most performed during pregnancy in Spain, thus failing to capture women not attending reproductive health care services (Organización Mundial de la Salud, 2016). Thus, these cases are often identified through sexual contact notification and particularly through opportunistic testing and screening. However, these tests are not always easily and universally available to the population (Toskin et al., 2020).

In addition, patterns of sexual behavior can be explained by social determinants such as gender, ethnicity, and immigration, which serve as axes of social inequalities in health (Borrell et al., 2013; Unemo et al., 2017). In this regard, the country of birth is understood as a structural variable associated with economic policies and the broader social culture, which helps explain the influence of inequalities in the daily lives of the population (Borrell et al., 2013). Some migrants are exposed to sexual vulnerability due to their migration route, social isolation, administrative and housing insecurity, and homophobia discrimination (Cordel et al., 2022). Indeed, research carried out in Spain amply shows that the risk of having an STI is higher in the migrant population (Diaz et al., 2013; Pérez-Morente et al., 2020).

Unlike infection with viral hepatitis A and B, in which past episodes confer immunity, infection by gonorrhea, syphilis or lymphogranuloma venereum (LGV) does not lead to immunity, and consequently affected individuals can have repeat episodes of these STI (Díez & Díaz, 2011). In other infectious diseases, such as tuberculosis, the reinfection rate is higher in persons living in areas with a high incidence of the infection (Millet et al., 2013). Thus, sexual networks, which can be defined as the ways and locations through which individuals connect with their sexual partners, may contribute to disparities in STI (MacCarthy et al., 2015). Sexual networks differ within and between societies, determining the potential for STI epidemics and opportunities for prevention (Morris et al., 2007). Therefore, strengthening efforts that could aid early diagnosis in persons at higher risk of reinfection could play a crucial role in STI and HIV transmission (Gesink et al., 2011). Moreover, to prevent a high frequency of reinfection, clinical practice guidelines recommend monitoring STIs not conferring immunity at 3 months after diagnosis (Sanidad et al., 2017).

Identifying the population groups most at risk of repeat STIs allows for the development of specific programs and interventions to promote sexual health and reduce serious reproductive health sequelae, antibiotic resistance and the burden of care on the health system (Tuddenham et al., 2022). However, there has been no prior population-based research on repeat STIs in our setting. Therefore, the aim of this study was to describe the epidemiological characteristics of persons with repeat bacterial STI (gonorrhea, syphilis or LGV) and to determine the factors associated with the risk of acquiring these infections in persons living in Barcelona according to their place of birth from 2007 to 2018.

## Method

This study was conducted in Barcelona (Catalonia, Spain), an urban area, whose census population consisted of 1,620,343 inhabitants in 2018 (Generalitat de Catalunya, 2022). In Catalonia, the reporting of all cases of gonorrhea, syphilis and LGV infection has been mandatory since 2007. Since that year, our mandatory disease surveillance system has no data on other bacterial infections, such as chlamydia (which was introduced in 2017).

### Subjects

This was a retrospective, cohort, population-based study in the city of Barcelona. We analyzed data from persons older than 14 years, living in the city, and diagnosed with gonorrhea, LGV infection, or syphilis (primary, secondary early latent or indeterminate) included in the STI registry of the Public Health Agency of Barcelona from January 2007 to December 2018. Case definitions were established following European standards (Salut et al., 2010). We excluded cases notified as late latent syphilis.

### Measures

Repeat STI (R-STI) were defined as a new episode of gonorrhea, syphilis or LGV infection in persons with a prior diagnosis of one of these infections. When the cause was the same in both episodes, time criteria were used to determine the second episode. Thus, in gonorrhea, R-STI was defined as an episode occurring at least 14 days after a prior treated episode. In LGV, the interval between the two episodes had to be at least 45 days. In primary syphilis, R-STI was defined as a second episode occurring 1 month after the previous treated episode. If the first diagnosis was secondary syphilis, R-STI was defined as follows: (1) a diagnosis of primary syphilis regardless of the time interval between the two episodes, (2) a diagnosis of secondary syphilis if there was a minimum time interval of at least 2 months between the two episodes, and (3) a diagnosis of early latent syphilis if there was a minimum time interval of at least 3 months between the two episodes. When the first diagnosis was early latent syphilis, repeat infection was defined as: (1) a diagnosis of primary or secondary syphilis regardless of the time interval between the two episodes, and (2) another diagnosis of early latent syphilis if there was an interval of 6 months or more after the prior treated episode. Cases of indeterminate syphilis were only classified as R-STI when the first diagnosis was something other than syphilis. Coinfection was defined as diagnosis of two or more STIs (syphilis, gonorrhea and/or LGV) on the same date or 6 days apart.

We analyzed the following sociodemographic variables: sex, age (< or ≥ 35 years, the same stratification used in the public policies of Barcelona to differentiate between the younger and older groups), place of birth (Spain/abroad), educational level (no education, primary school, secondary school, university education), district of residence according to the distribution of the family socioeconomic index in Barcelona (low/middle/high) (Ajuntament de Barcelona, 2017) and sexual behavior (heterosexual, gay, bisexual and other men who have had sex with men and transsexual or transgender women assigned male sex at birth [GBMSM/Trans]). Clinical variables consisted of coinfection with other STIs and HIV serostatus at the first diagnosis.

### Statistical Analysis

We carried out a descriptive analysis of the epidemiological characteristics of all persons diagnosed with STI—syphilis, gonorrhea, and LGV infections. The incidence of R-STI was calculated in person/years of follow-up. In persons with R-STI, length of follow-up was calculated as the differences in months between the first and second diagnosis. In persons without repeat infections, length of follow-up was calculated as the difference between the date of censure (December 31, 2018) and the date of diagnosis.

Kaplan–Meier estimates were used to calculate the time to the second episode for the different study variables and the log–rank test was used to determine the statistical significance of any differences. Cox regression models with 95% confidence intervals (CI) were used to compare variables between the two groups (R-STI and a single STI). Finally, a multivariate model was constructed with the variables that were significant in the bivariate analysis (*p*-value < .05) or those that were epidemiologically relevant. The variance inflation factor was calculated to detect multicollinearity. This model was stratified by place of birth to assess the differences. All analyses were performed using STATA (V.13; Stata Corporation, College Station, TX, USA).

## Results

### Characteristics of the Study Population

From 2007 to 2018, we detected 9927 persons with at least one diagnosis of a bacterial STI. Of these, there were 7640 (59.4%) episodes of gonorrhea, 4809 (36.1%) episodes of syphilis, and 863 (6.5%) of LGV, with a total of 13,312 episodes.

Of the total, 1690 persons (17.0%) had more than one episode, and 6.4% of these had more than two episodes. The average age of these groups was 33 (interquartile range [IQR]: 28–39) years. In all, 98.7% were men, 88.5% were GBMSM/Trans, and 54.2% were born in Spain. The incidence of R-STI was 4.5 [95% CI 4.31–4.81] cases/100 person-years of follow-up. According to each STI, the incidence of R-STI was 0. 59 [95% CI 0.52–0.67] cases/100 person-years for syphilis, 2.04 [95% CI 1.90–2.20] cases/100 person-years for gonorrhea and 0.22 [95% CI 0.17–0.27] cases/100 person-years for LGV. Table 1 summarizes the epidemiological characteristics of the study population by the presence or absence of R-STI.

**Table 1:** Epidemiological characteristics of persons diagnosed with gonorrhea, syphilis, and/or lymphogranuloma venereum (LGV) by the presence of repeat infection

	Repeat STI		Total (%)
	Yes (%)	No (%)	
Overall	1690 (100)	8237(100)	9927 (100)
*Sex assigned at birth*			
Male	1668 (98.7)	7101 (86.2)	8769 (88.3)
Female	22 (1.3)	1136 (13.8)	1158 (11.7)
*Age*			
34 years or less	990 (58.6)	4846 (58.8)	5836 (58.8)
35 years or more	700 (41.4)	3391 (41.2)	4091 (41.2)
*Country of birth*			
Abroad	718 (42.5)	3151 (38.3)	3869 (39.0)
Spain	916 (54.2)	3693 (44.8)	4609 (46.4)
Missing	56 (3.3)	1393 (16.9)	1449 (14.6)
*Educational level*			
None/Primary school	221 (13.1)	742 (9.0)	963 (9.7)
Secondary school	438 (25.9)	1491 (18.1)	1929 (19.4)
University	533 (31.5)	1615 (19.6)	2148 (21.6)
Missing	498 (29.5)	4389 (53.3)	4887 (49.2)
*District of residence by family income index*			
Very low-income/low-income districts	214 (12.7)	1414 (17.2)	1628 (16.4)
Lower middle/middle-income districts	740 (43.8)	3285 (39.9)	4025 (40.5)
High/very high-income districts	733 (43.4)	3045 (37.0)	3778 (38.1)
Missing	3 (0.2)	493 (6.0)	496 (5.0)
*Sexual behavior*			
Heterosexual	97 (5.7)	2040 (24.8)	2137 (21.5)
GBMSM/Trans*	1495 (88.5)	4265 (51.8)	5760 (58.0)
Missing	98 (5.8)	1932 (23.5)	2030 (20.4)
*First diagnosed infection*			
Syphilis	668 (39.5)	2826 (34.3)	3494 (35.2)
Gonorrhea	850 (50.3)	5129 (62.3)	5979 (60.2)
LGV	172 (10.2)	282 (3.4)	454 (4.6)
*HIV serostatus*			
Positive	743 (44.0)	1430 (17.4)	2173 (21.9)
Negative	947 (56.0)	6807 (82.6)	7754 (78.1)
*Coinfection with other STI at diagnosis*			
Yes	63 (3.7)	119 (1.5)	182 (1.8)
No	1627 (96.3)	8118 (98.6)	9745 (98.2)

Barcelona, 2007–2018

*STI* sexually transmitted infection,* GBMSM* Gay, bisexual and other men who have sex with men, Trans* is a transsexual o transgender woman who was assigned male at birth,* LGV* lymphogranuloma venereum

All women with R-STI (1.3%) had had sexual relations with men. Of them, 16 (72.7%) had been born abroad: nine in Latin America, two in western Europe, three in eastern Europe and two in eastern Asia. None of them had HIV coinfection or coinfection with another STI at the time of the first diagnosis.

**Fig. 1:**
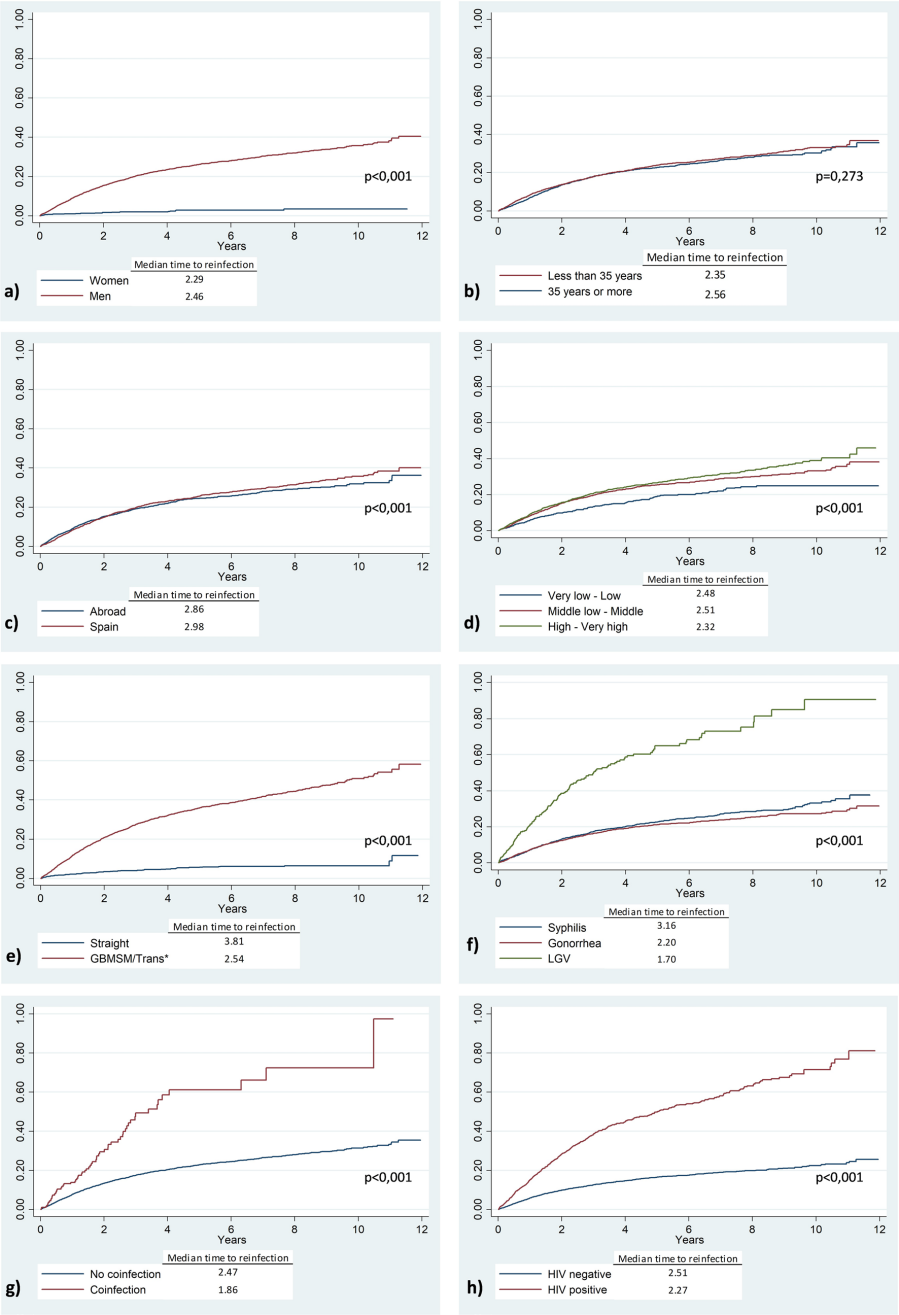
Time free from repeat STI in diagnosed cases of gonorrhea, syphilis and/or LGV by: **a** sex, **b** age, **c** place of birth, **d** place of residence by family income, **e** sexual behavior, **f** ethology of first STI diagnosis, **g** HIV serostatus, and **h** STI coinfection. Barcelona, 2007–2018

In the Kaplan–Meier analysis, the median time to R-STI was 30.5 (IQR: 14.1–68.1) months. The variables significantly associated with R-STI were: sex assigned at birth (*p*-value: .001), place of birth (*p*-value: .001), district of residence according to family index distribution (*p*-value: .001), sexual behavior etiology of the first diagnosis (*p*-value: .001), HIV coinfection (*p*-value: .001), and coinfection with other STI (*p*-value: .001) (Fig. 1). Table 2 shows the results of the univariate Cox analysis overall and stratified by birthplace. The risk of R-STI was higher in Spanish-born men than in foreign-born men (16.57 vs. 6.93). Comparison of other variables showed that the risk was higher in foreign-born persons with university studies (1.17 vs 1.40), those living in high/very high-income districts (1.42 vs 1.65) and in GBMSM /Trans (5.70 vs 7.49).

**Table 2:** Factors associated with repeat STI in diagnosed cases of gonorrhea, syphilis, and/or lymphogranuloma venereum (LGV)

Variable	Overall (HR)	Spain (HR)	Abroad (HR)
	(95%CI)	(95%CI)	(95%CI)
*Sex assigned at birth*			
Female	1	1	1
Male	9.98 (6.56–15.21)	16.57 (7.42–36.99)	6.93 (4.22–11.38)
*Age*			
35 years or more	1	1	1
34 years or less	1.03 (0.93–1.13)	1.11 (0.97–1.26)	0.97 (0.84–1.13)
*Educational level*			
None/Primary school	1	1	1
Secondary school	1.07 (0.91–1.27)	1.06 (0.85–1.31)	1.07 (0.84–1.37)
University	1.28 (1.10–1.51)	1.17 (0.95–1.46)	1.40 (1.11–1.77)
Missing	0.59 (0.51–0.69)	0.60 (0.48–0.75)	0.71 (0.56–0.90)
*Country of birth*			
Abroad	1	–	–
Spain	1.06 (0.96–1.17)	–	–
Missing	0.32 (0.24–0.42)		
*District of residence by family income index*			
Very low/low–income districts	1	1	1
Low-middle/middle-income districts	1.38 (1.19–1.61)	1.45 (1.19–1.76)	1.36 (1.05–1.76)
High/very high-income districts	1.53 (1.32–1.78)	1.42 (1.17–1.73)	1.65 (1.28–2.15)
Missing	0.04 (0.01–0.13)	0.05 (0.01–0.19)	0.05 (0.01–0.34)
*Sexual behavior*			
Heterosexual	1	1	1
GBMSM/Trans*	6.53 (5.32–8.02)	5.70 (4.33–7.51)	7.49 (5.51–10.45)
Missing	1.35 (1.02–1.79)	1.16 (0.78–1.73)	1.18 (0.69–2.03)
*First infection diagnosed*			
Syphilis	1	1	1
Gonorrhea	0.88 (0.79–0.98)	0.89 (0.78–1.03)	0.99 (0.85–1.16)
LGV	2.72 (2.30–3.22)	2.76 (2.21–3.44)	3.03 (1.92–3.23)
*HIV serostatus*			
Negative	1	1	1
Positive	3.03 (2.75–3.34)	2.88 (2.53–3.29)	2.93 (2.62–3.51)
*Coinfection with other STI at diagnosis*			
No	1	1	1
Yes	2.53 (1.97–3.26)	2.57 (1.77–3.72)	2.31 (1.64–3.26)

Univariate Cox model overall and stratified by place of birth. Barcelona. 2007–2018

*HR* Hazard ratio,* GBMSM* Gay, bisexual and other men who have sex with men, Trans* is a transsexual o transgender woman who was assigned male at birth,* LGV* lymphogranuloma venereum,* STI* sexually transmitted infection

On multivariate analysis, the factors independently associated with an increased risk of R-STI in the overall results were male sex assigned at birth (aHR: 3.45, 95% 2.22–5.36), age 34 years or less at the first diagnosis (aHR: 1.22, 95% 1.10–1.35), GBSMS/Trans (aHR: 4.03, 95% 3.24–5.03), a first infection with gonorrhea (aHR: 1.49, 95% 1.34–1.66) or LGV (aHR: 1.75, 95% 1.47–2.08) and being HIV-positive (aHR: 1.98, 95% 1.78–2.21) (Table 3). In contrast, the socioeconomic level of the district of residence lost statistical significance. Models stratified by place of birth showed similar differences in risk to the overall model, except for sex assigned at birth. Thus, the risk of R-STI was 6.6 times higher in men born in Spain than in women and was 1.8 times higher in men born abroad than in women. The other independent variables were similar in the two groups (Table 3).

**Table 3:** Factors associated with repeat STI in diagnosed cases of gonorrhea, syphilis, and lymphogranuloma venereum (LGV)

Variable	Overall	Spain	Abroad
	aHR (95%CI)	aHR (95%CI)	aHR (95%CI)
*Sex assigned at birth*			
Female	1	1	1
Male	3.45 (2.22–5.36)	6.57 (2.90–14.92)	1.91 (1.11–3.27)
*Age*			
35 years or more	1	1	1
34 years or less	1.22 (1.10–1.35)	1.29 (1.12–1.48)	1.18 (1.01–1.38)
*District of residence by family income index*			
Very low/low-income districts	1	1	1
Low-middle/middle-income districts	1.04 (0.89–1.21)	1.11 (0.91–1.35)	0.97 (0.74–1.25)
High/very high-income districts	1.03 (0.88–1.20)	1.01 (0.83–1.23)	1.02 (0.78–1.32)
Missing	0.04 (0.01–0.12)	0.04 (0.01–0.16)	0.04 (0.01–0.31)
*Sexual behavior*			
Heterosexual	1	1	1
GBMSM/Trans*	4.03 (3.24–5.03)	3.49 (2.61–4.67)	4.89 (3.46–6.94)
Missing	1.10 (0.82–1.47)	0.82 (0.55–1.22)	0.90 (0.52–1.57)
*First infection diagnosed*			
Syphilis	1	1	1
Gonorrhea	1.49 (1.34–1.66)	1.46 (1.26–1.69)	1.56 (1.32–1.83)
LGV	1.75 (1.47–2.08)	1.84 (1.47–2.32)	1.63 (1.25–2.13)
*HIV serostatus*			
Negative	1	1	1
Positive	1.98 (1.78–2.21)	1.99 (1.71–2.30)	2.02 (1.72–2.38)
* STI coinfection at first diagnosis*			
No	1	1	1
Yes	1.32 (1.02–1.71)	1.46 (1.00–2.12)	1.23 (0.87–1.75)

Multivariate cox model stratified by place of birth. Barcelona. 2007–2018

*HR* Hazard ratio,* GBMSM* Gay, bisexual and other men who have sex with men, Trans* is a transsexual o transgender woman who was assigned male at birth,* LGV* lymphogranuloma venereum,* STI* sexually transmitted infection

## Discussion

In this population-based study, 17.0% of persons diagnosed with gonorrhea, syphilis, and/or LGV in Barcelona had an R-STI in an 11-year period. The incidence rate of R-STI was 4.5 cases/100 person-years of follow-up. The factors associated with R-STI were male sex assigned at birth, age 34 years or less, GBMSM/Trans, a first diagnosis of gonorrhea or LGV, and HIV infection.

A study performed in Lima (Peru), which analyzed syphilis reinfections exclusively over a 2-year period, reported a reinfection rate of 7.5% (Park et al., 2016). In a cohort followed up in Wellington (New Zealand) for 3 years, the percentage of chlamydia and gonorrhea reinfection was 18%, and reinfection was more frequent in men of Māori and Pacific ethnicities (Rose et al., 2017). In general, it is difficult to compare these figures with our findings because most of these studies were conducted in populations infected with one or two STI, in shorter study periods than our own, and were not population-based. To our knowledge, the only population-based study published to date was conducted in Massachusetts (USA), where the proportion of repeat syphilis, gonorrhea, and chlamydial infections reported to the state surveillance system within a 2-year period was 14.2% (Hsu et al., 2018). These results highlight the need to increase studies on R-STI and calculate their incidence to allow comparison of data.

Our finding of an association between R-STI and HIV infection has previously been reported in other studies on R-STI conducted in Belgium and Brazil (Kenyon et al., 2014; Valeria et al., 2017). An explanation is that persons with HIV infection who undergo periodic screening have a higher likelihood of being diagnosed with asymptomatic STI (Kenyon et al., 2014).

This study found that younger patients (aged 34 years or less) had a 25% higher risk of R-STI than older patients. In line with these results, other studies have also reported that R-STI are more common in young persons (Trecker et al., 2015). This is a major public health problem, since having an STI while young has been associated with a threefold higher risk of acquiring HIV infection in adulthood (Newbern et al., 2013). Therefore, sexual and emotional health programs are required from an early age (including kindergarten level). The most effective programs have demonstrated that preventing STI and unintended pregnancies goes beyond focusing solely on sexual behavior. It is crucial address broader issues such as identity, sexual diversity, social justice, support for social–emotional learning, improved communication skills, awareness of gender stereotypes and sexual rights, and awareness of discrimination and oppression based in other social axes, such as racism and classism) (Goldfarb & Lieberman, 2021).

Regarding the sexual behavior of GBSMS/Trans, our data show an increased risk of R-STI (HR: 4.03; 95%CI 3.24–5.03). The increase in STI can be explained in the first years of the study, among other factors, by the adoption of HIV prevention strategies, such as seroadaptation, leading to practices such as serosorting (Purcell et al., 2017). Likewise, studies using the gender perspective have shown that men need to demonstrate that they are sexually active to respond to hegemonic models of masculinity, leading them to maintain risky practices in general, including in their sexual behavior (Jacques-Aviñó et al., 2018).

R-STI in GBMSM can also be explained by a decrease in condom use associated with drug consumption, the greater use of mobile apps to find sexual partners and the higher prevalence of mental and emotional health problems such as depression, which have previously been reported in this population (Jacques-Aviñó et al., 2018; Traeger et al., 2018). In addition, pre-exposure prophylactic (PrEP) treatment can increase risky sexual behaviors by encouraging an “optimistic perception” of a reduced risk of acquiring HIV, thus increasing the incidence of STI, as reported by previous studies (Serpa et al., 2020; Traeger et al., 2018).

A previous study reported that the incidence of the various STI in the city of Barcelona was higher in the foreign-born population (Salut & Barcelona, 2016). However, in our study, having an R-STI was associated with having been born in Spain. This finding was probably due to the fact that underdiagnosis of HIV is more common in the immigrant GBMSM population in Catalonia, suggesting that these individuals have less access to STI screening (Reyes-Urueña et al., 2018). Moreover, previous studies have shown that the immigrant population faces a higher number of barriers to accessing health services, experiences greater discrimination and has other social problems that increase their vulnerability (Wayal et al., 2017).

In our study, there were no substantial differences in the distribution of persons with R-ITS by district of residence. In contrast, other studies have reported that populations residing in disadvantaged neighborhoods and belonging to ethnic minorities had a higher prevalence of STI (Lutfi et al., 2018). In addition, our results could have been influenced by a greater geographical mobility among the migrant population within Spain and to other countries (Izquierdo et al., 2015).

In terms of prevention of R-STI episodes, several studies have reported that notifying sexual partners of an STI decreases the probability of transmission and reinfection (De et al., 2015). In addition, women tend to respond better than men to screening through reminder text messages (Izquierdo et al., 2015). In contrast, if only GBMSM are taken into account, the young population is less receptive to undergoing STI/HIV tests through an invitation sent through a sexual contact app (Gutiérrez et al., 2018). This situation poses major challenges to STI prevention among the young MSM population. We believe that important strategies are educational interventions in the school system and partner counseling. It is also crucial to involve primary care staff to maximize the impact of prevention efforts. Indeed, it has been demonstrated that health staff who develop counseling skills could reduce new infections in persons with a first diagnosis of STI (Wynn et al., 2019). There is also evidence that sexual health counseling and testing at primary care HIV consultations increase the likelihood of STI diagnosis (Mathé et al., 2023).

### Limitations

This study had some limitations. We were able to include only infections diagnosed in persons living in the city and were consequently unable to include any reinfections in persons who moved away during the study period. We were unable to identify cases of STI infection before 2007 as there was no registry. The incidence of R-STI could therefore be underestimated. In addition, cases in women might be under-notified, due to the high proportion of asymptomatic STI in this population. In contrast, cases of STI in persons with HIV, as well as those in GBMSM in general, might be overestimated, since these individuals usually have greater access to screening, despite being asymptomatic. Another limitation is that we did not have specific data to differentiate between identity and sexual orientation. For this reason, we followed classical epidemiological criteria and created a single category that includes gay men, bisexuals, and transwomen, assuming that the risks are similar (Nguyen et al., 2022).

### Conclusion

This study found a higher risk of R-STI in young people, GBMSM/Trans, persons with HIV infection, and those with a first diagnosis of gonorrhea and especially LGV. To prevent R-STI, we suggest strengthening programs specifically targeting these groups in the young population diagnosed with an STI. This would entail the active involvement of education and health professionals in prevention and sexual–emotional health promotion. Moreover, such programs should integrate social and behavioral determinants starting with school-aged teenagers. The programs should also consider the needs of this population from the perspective of sex-gender identity diversity and use an intercultural approach.

## Data Availability

Due to Spanish and EU data protection regulations, original register data must be requested from the respective registers.

## References

[CR29] Agència de Salut Pública de Barcelona. (2017). *La Salut a Barcelona 2016*. https://www.aspb.cat/wp-content/uploads/2017/11/Informe_Salut_2016.pdf

[CR2] Ajuntament de Barcelona. (2017). *Distribució Territorial de La Renda Familiar Disponible per Càpita a Barcelona 2017*; 2017. https://bcnroc.ajuntament.barcelona.cat/jspui/handle/11703/112233

[CR3] Borrell C, Pons-Vigués M, Morrison J, Díez È (2013). Factors and processes influencing health inequalities in urban areas. Journal of Epidemiology and Community Health.

[CR4] Carrere, J., Sánchez-Ledesema, E., Pericas, C., Cortés, M., Pérez, K., Artazcoz, L. (2023). *Salut i Drets Sexuals i Reproductius a Barcelona Any 2021*.

[CR6] Cordel H, Tantet C, Stempak T, Billaud E, Mosnier E, Huber F, Florence S, Leclerc D, Freire-Maresca A, de Champs Léger H, Ahouanto M, Linard F, Petruzzi M, Hamel E, Le Lay E, Lydié N, Simon A, Alcouffe L, Vignier N (2022). Addressing sexuality and sexual health with migrants. Practice guidelines. Infectious Disease Now.

[CR30] Departament de Salut. (2010). Direcció General de Salut Pública. *Definició de Cas de Les Malalties de Declaració Obligatòria*. 2010.

[CR8] Diaz, A.,, Garriga, C., Varela. J. A., Fernández, E., Sanz, I., Boronat, J., Gual, F., Colomo, C., de Munain, J. L., Esteban, V., Junquera, M. L., Martínez, B., Pueyo, I., Suárez, J., Barberá, M. J., Arando, M., Ureña, J. M., & Diez, M. (2013). Gonorrhoea diagnoses in a network of STI clinics in Spain during the period 2006–2010: Differences by sex and transmission route. *BMC Public Health, 13*(1). 10.1186/1471-2458-13-1093 10.1186/1471-2458-13-1093 PMC4222879 24274101

[CR9] Díez M, Díaz A (2011). Infecciones de transmisión sexual: Epidemiología y control. Revista Española de Sanidad Penitenciaria.

[CR7] Garcia de Olalla P, Molas E, Barberà MJ, Martín S, Arellano E, Gosch M, Saladie P, Carbonell T, Knobel H, Diez E, Caylà JA (2015). Effectiveness of a pilot partner notification program for new HIV cases in Barcelona, Spain. PLoS ONE.

[CR5] Generalitat de Catalunya. (2022). *Barcelona. El municipi en xifres*. Institut d’Estadística de Catalunya.

[CR10] Gesink DC, Sullivan AB, Miller WC, Bernstein KT (2011). Practice of epidemiology sexually transmitted disease core theory: Roles of person, place, and time. American Journal of Epidemiology.

[CR11] Goldfarb ES, Lieberman LD (2021). Three decades of research: The case for comprehensive sex education. Journal of Adolescent Health.

[CR1] Gutiérrez MA, Quevedo MF, Valle SM, Jacques-Aviñó C, David ED, Caylà JA, De Olalla PG (2018). Acceptability and effectiveness of using mobile applications to promote HIV and other STI testing among men who have sex with men in Barcelona, Spain. Sexually Transmitted Infections.

[CR12] Hsu KK, Molotnikov LE, Roosevelt KA, Elder HR, Klevens RM, DeMaria A, Aral SO (2018). Characteristics of cases with repeated sexually transmitted infections, Massachusetts, 2014–2016. Clinical Infectious Diseases.

[CR13] Izquierdo, M., Jimeno, J.F., Lacuesta, A. (2015). *Spain: From immigration to emigration?* Banco de Espana Working Paper No. 1503. 10.2139/ssrn.2566723

[CR14] Jacques-Aviñó C, García de Olalla P, González Antelo A, Fernández Quevedo M, Romaní O, Caylà JA (2018). The theory of masculinity in studies on HIV. A systematic review. Global Public Health.

[CR15] Kenyon C, Lynen L, Florence E, Caluwaerts S, Vandenbruaene M, Apers L, Soentjens P, Van Esbroeck M, Bottieau E (2014). Syphilis reinfections pose problems for syphilis diagnosis in Antwerp, Belgium—1992 to 2012. Eurosurveillance.

[CR16] Lutfi K, Jo M, Kristopher T, Gladys PF, Gladwin H (2018). Racial residential segregation and STI diagnosis among non-Hispanic Blacks, 2006–2010. Journal of Immigrant and Minority Health.

[CR17] MacCarthy S, Mena L, Chan PA, Rose J, Simmons D, Riggins R, Hoffmann M, Perez-Brumer A, Chamberlain N, Nunn A (2015). Sexual network profiles and risk factors for STIs among African-American sexual minorities in Mississippi: A cross-sectional analysis. LGBT Health.

[CR18] Mathé PJG, Usadel S, Rieg S, Kern WV, Müller MC (2023). Long-term follow-up after introduction of a systematic sexually transmitted infection screening program for men having sex with men living with HIV in a primary care setting: uptake, STI incidence, and risk factors for infection and reinfection. Infection.

[CR19] Millet, J.-P., Shaw, E., Orcau, À., Casals, M., Miró, J. M., & Caylà, J. A. (2013). Tuberculosis recurrence after completion treatment in a European city: Reinfection or relapse? *PLoS ONE,**8*(6). 10.1371/journal.pone.0064898 10.1371/journal.pone.0064898 PMC3679149 23776440

[CR31] Ministerio de Sanidad Servicios Sociales e Igualdad. (2017). *Documento de Consenso Sobre Diagnóstico y Tratamiento de Las Infecciones de Transmisión Sexual En Adultos, Niños y Adolescentes (Marzo 2017)*.

[CR20] Morris M, Goodreau S, Moody J, Holmes KK, Sparling PF, Stamm WE, Piot P, Wasserheit JN, Corey L, Cohen MS (2007). Sexual networks, concurrency, and STD/HIV. Sexually transmitted diseases.

[CR22] Newbern EC, Anschuetz GL, Eberhart MG, Salmon ME, Brady KA, De Los Reyes A, Baker JM, Asbel LE, Johnson CC, Schwarz DF (2013). Adolescent sexually transmitted infections and risk for subsequent HIV. American Journal of Public Health.

[CR23] Nguyen H, Hampel B, Garcia Nuñez D, Battegay M, Hachfeld A, Bernasconi E, Calmy A, Cavassini M, Vernazza P, Fellay J, Rudolph H, Huber M, Leuzinger K, Perreau M, Scherrer A, Ramette AN, Yerly S, Günthard HF, Kouyos RD, Kusejko K, Swiss HIV Cohort Study (2022). Identifying and characterizing trans women in the Swiss HIV Cohort Study as an epidemiologically distinct risk group. Clinical Infectious Diseases.

[CR21] Organización Mundial de la Salud. (2016). *Estrategia Mundial Del Sector de La Salud Contra Las Infecciones de Transmisión Sexual 2016–2021*. http://apps.who.int/iris/bitstream/10665/250253/1/WHO-RHR-16.09-spa.pdf?ua=1&ua=1

[CR24] Park H, Konda KA, Roberts CP, Maguiña JL, Leon SR, Clark JL, Coates TJ, Caceres CF, Klausner JD (2016). Risk factors associated with incident syphilis in a cohort of high-risk men in peru. PLoS ONE.

[CR25] Pérez-Morente MÁ, Martín-Salvador A, Gázquez-López M, Femia-Marzo P, Pozo-Cano MD, Hueso-Montoro C, Martínez-García E (2020). Economic crisis and sexually transmitted infections: A comparison between native and immigrant populations in a specialised centre in Granada, Spain. International Journal of Environmental Research and Public Health.

[CR26] Purcell DW, Higa D, Mizuno Y, Lyles C (2017). Quantifying the harms and benefits from serosorting among HIV-negative gay and bisexual men: A systematic review and meta-analysis. AIDS and Behavior.

[CR27] Reyes-Urueña JM, Campbell CNJ, Vives N, Esteve A, Ambrosioni J, Tural C, Ferrer E, Navarro G, Force L, García I, Masabeu À, Vilaró JM, García de Olalla P, Caylà JA, Miró JM, Casabona J, PISCIS Investigators (2018). Estimating the HIV undiagnosed population in Catalonia, Spain: Descriptive and comparative data analysis to identify differences in MSM stratified by migrant and Spanish-born population. British Medical Journal Open.

[CR28] Rose, S. B., Garrett, S. M., Stanley, J., & Pullon, S. R. H. (2017). Retesting and repeat positivity following diagnosis of* Chlamydia trachomatis* and* Neisseria gonorrhoea* in New Zealand: A retrospective cohort study. *BMC Infectious Diseases,**17*(526). 10.1186/s12879-017-2635-y 10.1186/s12879-017-2635-y PMC5534075 28754106

[CR32] Serpa JA, Huynh GN, Nickell JB, Miao H (2020). Human immunodeficiency virus pre-exposure prophylaxis and increased incidence of sexually transmitted infections in the United States. Clinical Infectious Diseases.

[CR33] Toskin I, Govender V, Blondeel K, Murtagh M, Unemo M, Zemouri C, Peeling RW, Kiarie J (2020). Call to action for health systems integration of point-of-care testing to mitigate the transmission and burden of sexually transmitted infections. Sexually Transmitted Infections.

[CR34] Traeger MW, Schroeder SE, Wright EJ, Hellard ME, Cornelisse VJ, Doyle JS, Stoové MA (2018). Effects of pre-exposure prophylaxis for the prevention of HIV infection on sexual risk behavior in men who have sex with men: A systematic review and meta-analysis. Clinical Infectious Diseases.

[CR35] Trecker MA, Dillon JAR, Lloyd K, Hennink M, Waldner CL (2015). Demographic and behavioural characteristics predict bacterial STI reinfection and coinfection among a cross-sectional sample of laboratory-confirmed gonorrhea cases in a local health region from Saskatchewan, Canada. Canadian Journal of Public Health.

[CR36] Tuddenham S, Hamill M, Ghanem K (2022). Diagnosis and treatment of sexually transmitted infections: A review. Journal of the American Medical Association.

[CR37] Unemo M, Bradshaw CS, Hocking JS, de Vries HJC, Francis SC, Mabey D, Marrazzo JM, Sonder GJB, Schwebke JR, Hoornenborg E, Peeling RW, Philip SS, Low N, Fairley CK (2017). Sexually transmitted infections: Challenges ahead. Lancet Infectious Disease.

[CR38] Unidad de vigilancia del VIH y conductas de riesgo. (2019). *Vigilancia Epidemiológica de Las Infecciones de Transmisión Sexual En España, 2017*.

[CR39] Valeria CDA, Rita M, Donalisio MR, Cordeiro R (2017). Factors associated with reinfection of syphilis in reference centers for sexually transmitted infections. Revista da Saude Publica.

[CR40] Wayal S, Hughes G, Sonnenberg P, Mohammed H, Copas AJ, Gerressu M, Tanton C, Furegato M, Mercer CH (2017). Ethnic variations in sexual behaviours and sexual health markers: Findings from the third British National Survey of Sexual Attitudes and Lifestyles (Natsal-3). Lancet Public Health.

[CR41] Wynn A, Moucheraud C, Moshashane N, Offorjebe OA, Ramogola-Masire D, Klausner JD, Morroni C (2019). Using partner notification to address curable sexually transmitted infections in a high HIV prevalence context: A qualitative study about partner notification in Botswana. BMC Public Health.

